# Supplementary material for: Comparative inequalities in child dental caries across four countries: Examination of international birth cohorts and implications for oral health policy
Source: PLoS One. 2022 Aug 31;17(8):e0268899. doi: 10.1371/journal.pone.0268899 (PMC9432734; doi:10.1371/journal.pone.0268899)
Supplement: S1 Table — (DOCX) [file pone.0268899.s001.docx]

**S1 Table. Oral health policy and service mapping across countries.**

|  | **Australia (LSAC)** | **Quebec, Canada (QLSCD)** | **Rotterdam, Netherlands (Gen R)** | **Southeast Sweden (ABIS)** |
| --- | --- | --- | --- | --- |
| **Publicly funded oral health services** | Medicare, Australia’s publicly funded health system, provides limited oral health coverage.^1^  Public dental practices have long waiting lists^2^ and although state initiatives such as school dental services typically target children in primary school, the majority of preschool or high school students are left without public dental care. | Yes - The Régie de l’assurance maladie du Québec only covers a portion of expenses up to age 10 years;^3,4^ namely:   - one examination per year - emergency examinations - x-rays (including panoramic x-rays) - local or general anesthesia - amalgam (grey) fillings for posterior teeth - fillings using esthetic materials for anterior teeth - refabricated crowns | Yes. Public are required to take out a basic insurance package to cover costs of medical treatment. Children under the age of 18 are covered under their parent’s premium for the basic insurance package and dental care. Children under the age of 18 don’t pay for basic insurance.  Dental treatment for children below the age of 18 is reimbursed under the terms of the basic insurance package^5^ | Yes – free of charge from birth to 23 years of age  There is no cost for the first dental care visit and parents can choose whether to see a private or public dentist  The public dentistry in Sweden is predominantly (around 95-98%) responsible for dental care of school aged children. Each child will have a chosen dentist responsible for dental check-ups even in later school ages^6^ |
| **Out of pocket expenses** | Australians are often left with out of pocket expenses as the majority of dental reviews are self-funded or paid by a private health fund.^7^ Australians spent 19.6% of their health expenditure on dental services in 2016–2017.^7^ | Yes - in 2015, publicly financed programs covered about 6.3% of the dental service expenditures; private insurers (employment related and personal) covered 56.2%, and users paid out-of-pocket for the remaining 36.7% of expenses.^8^ | No | No |
| **Access to dental services** | *The Child Dental Benefits Schedule (CDBS)*, which is means tested, allows children aged between 2-17 years to benefit from basic dental services capped at $1000 over two years.^9^  Only 29% of eligible children utilised the program during its first year.^10^ | *Pediatric Dental Care Program* allows children under 10 years of age to free selected basic dental services  Irrespective of age, children from families receiving financial assistance for at least 12 consecutive months are also entitled to basic dental care; entitled care and services must be received from a dentist who participates in the Health Insurance Plan^11,12^ | Dental care is free for children under the age of 18 under the *Dutch health insurance (zorgverzekering)* | For children and young adults up to the age of 23 years all dental care is free including regular check-ups under *Swedish Public Social Security Insurance*^6^ |
| **Access to fluoridated water** | Australia mostly has very low levels of naturally occurring fluoride. However, community water fluoridation means that around 89% of Australians have access to fluoridated drinking water (within the range of 0.6 to 1.1 mg/L).^13^ | According to Statistics Canada (<https://www.canada.ca/en/services/health/publications/healthy-living/community-water-fluoridation-across-canada-2017.html>), 2.49% of the population in Québec has fluoridated systems and about 0.5% has naturally occurring fluoride in well water. | Dutch drinking water companies do not add fluoride to the drinking water. The natural concentration of fluoride in drinking water varies between 0.05 and 0.25 mg/L^14^ | Sweden has never had any added fluoridation in drinking water, but low levels of natural fluoridation can occur in different regions. |

Note: See S1 File for associated reference list.
