# Supplementary material for: Comparative inequalities in child dental caries across four countries: Examination of international birth cohorts and implications for oral health policy
Source: PLoS One. 2022 Aug 31;17(8):e0268899. doi: 10.1371/journal.pone.0268899 (PMC9432734; doi:10.1371/journal.pone.0268899)
Supplement: S2 Table — (DOCX) [file pone.0268899.s002.docx]

**S2 Table. Recommendations for the new WHO global strategy for oral health supported by key findings from this study.**

| **Recommendations of new WHO global strategy for oral health**^15^ | | **Relevant key findings from this study** |
| --- | --- | --- |
| **1. Inclusion and community engagement** | | |
|  | Include the diverse voices of people living with oral diseases in policy dialogues, programme planning, and evaluations to ensure that needs and views of disadvantaged populations are addressed when designing inclusive, accessible, and affordable oral health-care systems. | Population wide empirical findings of social gradients highlight the importance of focused studies to ensure inclusive policy and service provision. |
| **2. Place equity and social justice at the core** | | |
|  | Addressing oral health inequalities and their root causes must be central in all policies and future initiatives, fully aligned with the goals of primary health care and universal health coverage (UHC). | Supported by empirical findings of social gradients in dental caries according to income and maternal education. |
| **3. Tackle sugars as a major common risk factor** | | |
|  | The evidence of the negative impact of sugars on oral health provides an additional framing option to strengthen upstream population-wide measures, together with limiting the risks from other unhealthy foods and commodities as well as countering industry interference with oral health and non-communicable disease (NCD) strategies. | Supported by empirical findings of the negative impact of sugary diet on dental caries amongst children. |
| **4. Embrace major system reforms** | | |
|  | The integration of oral health care within UHC requires essential, cost-effective intervention packages, integrative delivery and financing models, and adaptations in educating oral health professionals. | Evident in the variability in dental health care policy and provision in the four countries and corresponding inequalities in dental caries amongst children. |
| **5. Better data for decision making** | | |
|  | Data-driven and evidence-informed policy decision making on oral health needs functioning monitoring and evaluation systems that are fully integrated with existing health monitoring and surveillance approaches. | Observational data analysis provided proof of concept that should be extended to health monitoring, surveillance (with direct dental caries measurement), and intervention studies to support decision making. |
| **6. Close financing gaps** | | |
|  | Oral health-care financing needs to be addressed as part of the overall NCD financing agenda, with special focus on increased public investments in low-income and middle-income countries, combined with reallocation of spending towards cost-effective best-buy interventions. | Evident in the variability in oral health care policy and provision in the four countries and corresponding inequalities in dental caries amongst children. |

Note: See S1 File for associated reference list.
