# Supplementary material for: Comparative inequalities in child dental caries across four countries: Examination of international birth cohorts and implications for oral health policy
Source: PLoS One. 2022 Aug 31;17(8):e0268899. doi: 10.1371/journal.pone.0268899 (PMC9432734; doi:10.1371/journal.pone.0268899)
Supplement: S1 File — (DOCX) [file pone.0268899.s003.docx]

**S1 File. Supplementary reference list for S1 Table and S2 Table.**

1. Australian Government Services Australia. Health care and Medicare: How you can get affordable health care and access our services. 2020; https://www.servicesaustralia.gov.au/individuals/subjects/whats-covered-medicare/health-care-and-medicare. Accessed 9 April 2020.

2. Australian Institute of Health and Welfare. *A discussion of public dental waiting times information in Australia: 2013-14 to 2016-17.* Canberra, Australia: Author; 2018.

3. Labrie Y. *The other health care system: Four areas where the private sector answers patients’ needs.* Canada: Montreal Economic Institute;2015.

4. Canadian Academy of Health Sciences. *Improving access to oral health care for vulnerable people living in Canada.* Ottawa, Canada: Author;2014.

5. Schuller A, van Dommelen P, Poorterman J. Trends in oral health in young people in the Netherlands over the past 20 years: A study in a changing context. *Community Dentistry and Oral Epidemiology.* 2014;42(2):178-184.

6. Swedish Public Dental Service. About the Swedish Public Dental Service. 2014; http://www.folktandvarden.se/in-english/about-the-swedish-public-dental-service/. Accessed 5 September 2019.

7. Australian Institute of Health and Welfare. *Health expenditure Australia 2016–17.* Canberra, Australia: Author;2018.

8. Canadian Dental Association. *The state of oral health in Canada.* Ontario, Canada: Author; 2017.

9. Department of Health. The Child Dental Benefits Schedule. 2014; http://www.health.gov.au/internet/main/publishing.nsf/content/childdental. Accessed 27 August 2018.

10. Department of Health. *Report on the third review of the Dental Benefits Act 2008.* Canberra, Australia: Author;2016.

11. Régie de l’assurance maladie du Québec. Dental services coverage. 2017; https://www.ramq.gouv.qc.ca/SiteCollectionDocuments/citoyens/en/depliants/depl-services-dentaires-en.pdf. Accessed 8 April 2020.

12. Shaw J, Farmer J. *An environmental scan of publicly financed dental care in Canada: 2015 update.* Canada: Saskatchewan Oral Health Coalition Inc.;2015.

13. National Health and Medical Research Council (NHMRC). *NHMRC Public Statement 2017: Water fluoridation and human health in Australia.* Canberra, Australia: Author; 2017.

14. Drink Water Platform. Fluoride in drinkwater: Alle vragen en antwoorden. 2020; https://www.drinkwaterplatform.nl/fluoride-in-drinkwater-alle-vragen-en-antwoorden/. Accessed 17 June 2020.

15. Benzian H, Guarnizo-Herreño CC, Kearns C, Muriithi MW, Watt RG. The WHO global strategy for oral health: an opportunity for bold action. The Lancet. 2021. doi: 10.1016/S0140-6736(21)01404-5.
