# Supplementary material for: Comparative inequalities in child dental caries across four countries: Examination of international birth cohorts and implications for oral health policy
Source: PLoS One. 2022 Aug 31;17(8):e0268899. doi: 10.1371/journal.pone.0268899 (PMC9432734; doi:10.1371/journal.pone.0268899)
Supplement: Attachment — Submitted filename: Response to reviewers 14.02.22.docx [file pone.0268899.s004.docx]

# Response to reviewers

## Editor comments

| 1 | Please ensure that your manuscript meets PLOS ONE's style requirements, including those for file naming. The PLOS ONE style templates can be found at  https://journals.plos.org/plosone/s/file?id=wjVg/PLOSOne_formatting_sample_main_body.pdf and  https://journals.plos.org/plosone/s/file?id=ba62/PLOSOne_formatting_sample_title_authors_affiliations.pdf. | We have carefully checked over the PLOS ONE style templates and ensured that our manuscript meets PLOS ONE’s style requirements. |
| --- | --- | --- |
| 2 | You indicated that you had ethical approval for your study. In your Methods section, please ensure you have also stated whether you obtained consent from parents or guardians of the minors included in the study or whether the research ethics committee or IRB specifically waived the need for their consent. | In Table 1, we now include the statement: “Explicit informed written consent was obtained for all participants (parents, guardians) across all cohorts”. |
| 3 | Please include your full ethics statement in the ‘Methods’ section of your manuscript file. In your statement, please include the full name of the IRB or ethics committee who approved or waived your study, as well as whether or not you obtained informed written or verbal consent. If consent was waived for your study, please include this information in your statement as well. . | The manuscript is based on a secondary analysis of previously collected birth cohort data. We have strived to articulate this clearly in the Methods section. Concordia University gave ethics approval for secondary use of the dataset. The original birth cohorts obtained informed written consent. |
| 4 | Please provide additional details regarding participant consent. In the Methods section, please ensure that you have specified (1) whether consent was informed and (2) what type you obtained (for instance, written or verbal). If your study included minors, state whether you obtained consent from parents or guardians. If the need for consent was waived by the ethics committee, please include this information. | All original birth cohorts complied with the ethical standards of their relevant institutional and/or national committees and with the Helsinki Declaration of 1964, and its later amendments. Information summarizing what participation would involve was provided in both oral and written form. Explicit informed written consent was obtained for all participants (parents, guardians). We have included greater details in the online submission information and in the Methods section.  Within the Data Sources section of the Materials and Methods section, we now include the statements: “Concordia University Human Research Ethics Committee certified the ethical acceptability for EPOCH’s secondary data use (#2011028). All original birth cohorts complied with the ethical standards of their relevant institutional and/or national committees and with the Helsinki Declaration of 1964, and its later amendments. Information summarizing what participation would involve was provided in both oral and written form.”  Please see also response to Editor comments #2 and #3 above. |
| 5 | Please state what role the funders took in the study.  If the funders had no role, please state: "The funders had no role in study design, data collection and analysis, decision to publish, or preparation of the manuscript."  If this statement is not correct you must amend it as needed.  Please include this amended Role of Funder statement in your cover letter; we will change the online submission form on your behalf. | In the Introduction, we now include the statement: “The EPOCH study, funded by the Canadian Institutes of Health Research (CIHR), was conceived and planned by researchers in the International Network for Research in Inequalities in Child Health (INRICH) [www.inrichnetwork.org]”.  We have included the suggested Role of Funder statement in our cover letter. |
| 6 | We note that you have indicated that data from this study are available upon request. PLOS only allows data to be available upon request if there are legal or ethical restrictions on sharing data publicly. For more information on unacceptable data access restrictions, please see http://journals.plos.org/plosone/s/data-availability#loc-unacceptable-data-access-restrictions.  In your revised cover letter, please address the following prompts:  a) If there are ethical or legal restrictions on sharing a de-identified data set, please explain them in detail (e.g., data contain potentially sensitive information, data are owned by a third-party organization, etc.) and who has imposed them (e.g., an ethics committee). Please also provide contact information for a data access committee, ethics committee, or other institutional body to which data requests may be sent.  b) If there are no restrictions, please upload the minimal anonymized data set necessary to replicate your study findings as either Supporting Information files or to a stable, public repository and provide us with the relevant URLs, DOIs, or accession numbers. For a list of acceptable repositories, please see http://journals.plos.org/plosone/s/data-availability#loc-recommended-repositories.  We will update your Data Availability statement on your behalf to reflect the information you provide. | We have included statements of Data Availability, Data Access, and Dual Publication in our cover letter.  In the Online Submission Information, we included a Data Availability Statement explaining that we are using data from third parties (i.e., original birth cohorts). We explicitly stated that we do not own these data nor do we have permission to distribute them. This statement is retained in the submitted revision. |
| 7 | One of the noted authors is a group or consortium [EPOCH Collaborative Group]. In addition to naming the author group, please list the individual authors and affiliations within this group in the acknowledgments section of your manuscript. Please also indicate clearly a lead author for this group along with a contact email address. | We have listed all individual authors from the EPOCH Collaborative Group and their affiliations in the acknowledgements section of the manuscript. We have also included the email address for Jennifer J. McGrath in the acknowledgements section as she is the email contact for the EPOCH Collaborative Group. |

## Reviewer 1

| 1 | The study discussed an important topic and its results are valuable to scientific community. | We thank the reviewer for their feedback. |
| --- | --- | --- |
| 2 | Introduction:  *page#4, Line 99 : Replace 'lower-income' by 'Low-income countries'. Also change 'higher-income' to 'high-income' | We have replaced the terms “lower-income” and “higher-income” with “low-income” and “high-income” throughout the manuscript. |
| 3 | *The aim of the study (objectives) should be written in a clear way. | We have revised the Aims paragraph. It now reads:  “Comparisons across these high-income jurisdictions/countries can provide rich insight into child dental caries prevalence, and associated health policies and social inequalities. This research is both timely and necessary to advance potential future policy action. The aim of the current study was to investigate comparative social gradients and associated risk factors for child dental caries using longitudinal cohort data across four high-income countries. Observing differential childhood caries outcomes according to household income and maternal education would provide evidence to determine whether childhood dental caries was experienced equitably within high income countries. Policy mechanisms that could explain any differences in the extent of inequalities, such as oral health care provision and water fluoridation, were then considered.” |
| 4 | *The rationale of the study & Null-hypothesis should be clearly stated | The aims and rationale for the study are now more clearly articulated in the Aims paragraph, as per our response to Reviewer 1 comment #3. |
| 5 | *Furthermore, the authors massively used adjectives (We/Our) which sometimes weakening the scientific structure, hence, the reviewer suggest to revise these sentences | We have revised the manuscript in light of this comment and removed all use of we/our. |
| 6 | Methodology :  *Table text citation should be done in a proper way (e.g Table 1, Table 2 , ......etc) | We have cited tables within text as per the “PLOS Manuscript Body Formatting Guidelines”. |
| 7 | Conclusions :  It looks like results , hence the reviewer suggests to revise. | We have revised the Conclusions section. It now reads:  “Clear gradients of social inequalities for child dental caries exist across four high income countries in this study. Variation in the prevalence of child dental caries and the extent of social inequalities across jurisdictions suggest that each may be responsive to oral health policy. The achievable combination of universal dental insurance and care (which includes adults/parents and children) and water fluoridation may be the necessary policy levers to achieve lower and more equitable rates of dental caries. To differentially benefit socio-economically disadvantaged groups of children, and sustain subsequent longer-term adult health benefits, it is timely to consider these universal and powerful public health measures in order to deliver on the aspirations of the WHO global oral health strategy.” |
| 8 | Overall comment about the manuscript language: It seems that this manuscript was written by native English speakers, however , the reviewer highly recommends revising the manuscript text by an expert academic writer to fulfill basic requirements of scientific writing | We have revised the entire manuscript and improved the language and writing style. This includes using past tense through the manuscript, removing the use of adjectives (as per Reviewer 1 comment #5), correcting grammatical errors, and revising any unclear statements. |

## Reviewer 2

| 1 | The current paper investigated inequalities in the prevalence of dental caries across four countries using birth cohorts. As the authors highlighted, dental caries although preventable still a burden especially among underserved and disadvantaged populations. Therefore there is an urgent need to investigate these inequalities and policies to address them. The paper is well written and the multi-centre methodology allows the reader to have a holistic view of different countries approaches. However, here are some points/concerns that the authors need to consider: | We thank the reviewer for their feedback. |
| --- | --- | --- |
| 2 | _ Title: the "Oral health policy" in the title may not be appropriate as this was not investigated nor was it part of the analysis but rather discussed in the context of the results. It might be beneficial if these policies were part of the variables investigated. | The title has been amended as follows to clarify the comparison made and consideration of implications for policy:  ‘Comparative inequalities in child dental caries across four countries: Examination of international birth cohorts and implications for oral health policy’  Please see the response to Reviewer 2 comment #8 for further discussion of the approaches taken to interpreting the potential influence of policies across the different jurisdictions included. |
| 3 | _ Introduction: The authors may consider summarizing the sections related to caries risk factors and elaborate on the caries prevalence in the four countries. Any previous studies on caries prevalence/risk factors? Also it is important to give an idea about current preventive programs/measures and policies, similar to what was mentioned about Australia. | The introduction has been extended to provide more of an international perspective on the prevalence, risk factors and policies relevant to children’s oral health and dental caries.  Specific prevalence estimates for the jurisdictions considered within the paper are limited within the literature and may be reliant on the studies analysed within the manuscript (e.g., the GenR study in the Netherlands[1]).  As such specific estimates of prevalence and risk factor associations are presented as the focus of the cross-jurisdictional analyses within the paper. |
| 4 | - Materials and Methods: |  |
| 4a | Please write the exact period of the study for each country. | This information related to Materials and Methods is now provided in Table 1. |
| 4b | From where was the participants recruited? hospitals or community centres? |  |
| 4c | What were the inclusion and exclusion criteria? |  |
| 4d | The participants were investigated/examined at single time or multiple time slots? please clarify. |  |
| 4e | For those who were examined, who conducted the examination? please provide details on the examination process and calibration. |  |
| 4f | For those whose parents reported on their caries: was that through an interview or questionnaire? |  |
| 4g | How was caries risk factors investigated ? If a questionnaire was used, why oral hygiene habits, dental visits or exposure to preventive measures investigated? | Caries risk factors were investigated where there had been a clear rationale identified in the literature (please see Introduction) and where suitable survey items were available in the cohorts included (please see further description in the Materials and Methods and Table 1).  As a secondary analysis of existing cohorts in which the questions had been specified in the original design, we were limited to using the information available rather than being able to design questions de-novo. |
| 5 | "Oral health provision is thus explored at a policy level by country/region" I still don't see how was this assessed in relation to study outcome. | The sentence now reads: “Oral health service provision is thus explored at a policy level by jurisdiction”.  The studies did not themselves collect information about local or national policies thus we didn’t have the policies as measured variables within the cohort data. In this instance we have interpreted the empirical cohort data and findings in context with the Author team’s expert knowledge of the policy context in each of the jurisdictions to draw the conclusions we have given.  In the cited Australian oral health manuscript by a number of the Authors[2], local data regarding water fluoridation were linked to the cohort data at a postcode level and used as measured variables. In contrast, given the differences in provision of water fluoridation, or not, these were interpreted at a jurisdictional level for this international comparison rather than at the local level.  The potential influences of different policies was considered at an interpretive level given the limited number of jurisdictions considered in this analysis (Australia; Québec, Canada; Rotterdam, the Netherlands; and Southeast Sweden). We note that with a larger number of jurisdictions it would be feasible to consider the approach of using meta-regression where data from different cohorts would be modelled with summary outcomes and variables indicating different policies included in the modelling. This type of technique would usually be used when there are a more substantive number of jurisdictions and the focus is primarily on the between jurisdictional differences rather than within cohort effects. |
| 6 | Please report on consent from patients. | Please see response to Editor comment #2. |
| 7 | Any information about children/mothers' medical health? | Each of the cohorts requests detailed information about other aspects of children/mothers’ health and medical conditions. Examination of inequalities in children’s health has been the subject of other manuscripts under submission by the EPOCH collaboration including ADHD, obesity, and chronic health conditions to date.  It is likely that other aspects of children/mothers’ medical health mediate the impact of socioeconomic disadvantage on dental caries. Aspects of health other than specific oral health behaviours have not been included to avoid potentially over-controlling the estimated effects of socioeconomic disadvantage and specific oral health behaviours. |
| 8 | -Results: |  |
| 8a | Please report on total number of participants for each country. Were there any loss of participants? It would be beneficial if the authors use a chart to clarify the recruitment process over time. | Additional details of the cohort participants and retention have been added to the summary of the cohort details (Table 1) and referenced in the description of the study methods and results. |
| 8b | The authors may consider re-arranging this section either by country and report findings under each or by variables investigated and report each country separately for more clarity. | The results section has been reviewed and revised to ensure the findings are presented accessibly. As the purpose of the analysis was to look comparatively across countries we have continued to integrate the description of the findings across countries within the results. Sub-headings have been added to indicate the variables of focus in each stage. |
| 9 | -Discussion: |  |
| 9a | - Table 3: It is uncommon using tables as part of the discussion, I believe instead it would be better if these points/recommendations are discussed in the context of the the evident provided and the study results. | We have moved this summary table into the Supplementary Information (S2 Table) and highlighted key points in the discussion in context with the study findings. The relevant paragraph in the Public Health Implications section now reads: “The study findings align and provide further empirical evidence supporting recent recommendations from the WHO global strategy for oral health.[3] Key elements of this strategy (summarized in S2 Table) include the need for major system reforms to provide inclusive, accessible, and affordable oral health-care including the need to close financing gaps to align goals of universal primary health care coverage; and establishing data-driven evidence-informed policy for oral health (including the importance of tackling risk factors such as sugar consumption) and health care. While the WHO recommendations are more focused on low- and middle-income countries, this study is both timely and necessary to advance potential policy action towards equitable oral health and provision of care within high-income jurisdictions.[3]”. |
| 10 | - Abstract: |  |
| 10a | Needs to be re-written in light of the concerns raised. | The abstract has been revised in light of the concerns raised by Reviewers to maximise clarity and accessibility of the findings. |
| 10b | - Please use STROBE checklist when revising the manuscript. | The STROBE checklist has been reviewed and resubmitted with the revised manuscript. |

**References**

1.	van der Tas J, Kragt L, Elfrink M, Bertens L, Jaddoe V, Moll H, et al. Social inequalities and dental caries in six-year-old children from the Netherlands. J Dent. 2017;62:18-24. Epub 04/24. doi: 10.1016/j.jdent.2017.04.008. PubMed PMID: 28450065.

2.	Goldfeld S, Francis K, Hoq M, Do L, O'Connor E, Mensah F. The impact of policy modifiable factors on inequalities in rates of child dental caries in Australia. International Journal of Environmental Research and Public Health. 2019;16(11):E1970. PubMed PMID: 31163687.

3.	Benzian H, Guarnizo-Herreño CC, Kearns C, Muriithi MW, Watt RG. The WHO global strategy for oral health: an opportunity for bold action. The Lancet. 2021. doi: 10.1016/S0140-6736(21)01404-5.
